# Supplementary material for: Sensitive extraction-free SARS-CoV-2 RNA virus detection using a chelating resin
Source: iScience. 2021 Aug 9;24(9):102960. doi: 10.1016/j.isci.2021.102960 (PMC8349732; doi:10.1016/j.isci.2021.102960)
Supplement: Document S1. Figures S1–S4 and Table S1 [file mmc1.pdf]

iScience, Volume *24*

Supplemental information

Sensitive extraction-free SARS-CoV-2 RNA virus detection using a chelating resin

Bin Guan, Karen M. Frank, José O. Maldonado, Margaret Beach, Eileen Pelayo, Blake M. Warner, and Robert B. Hufnagel

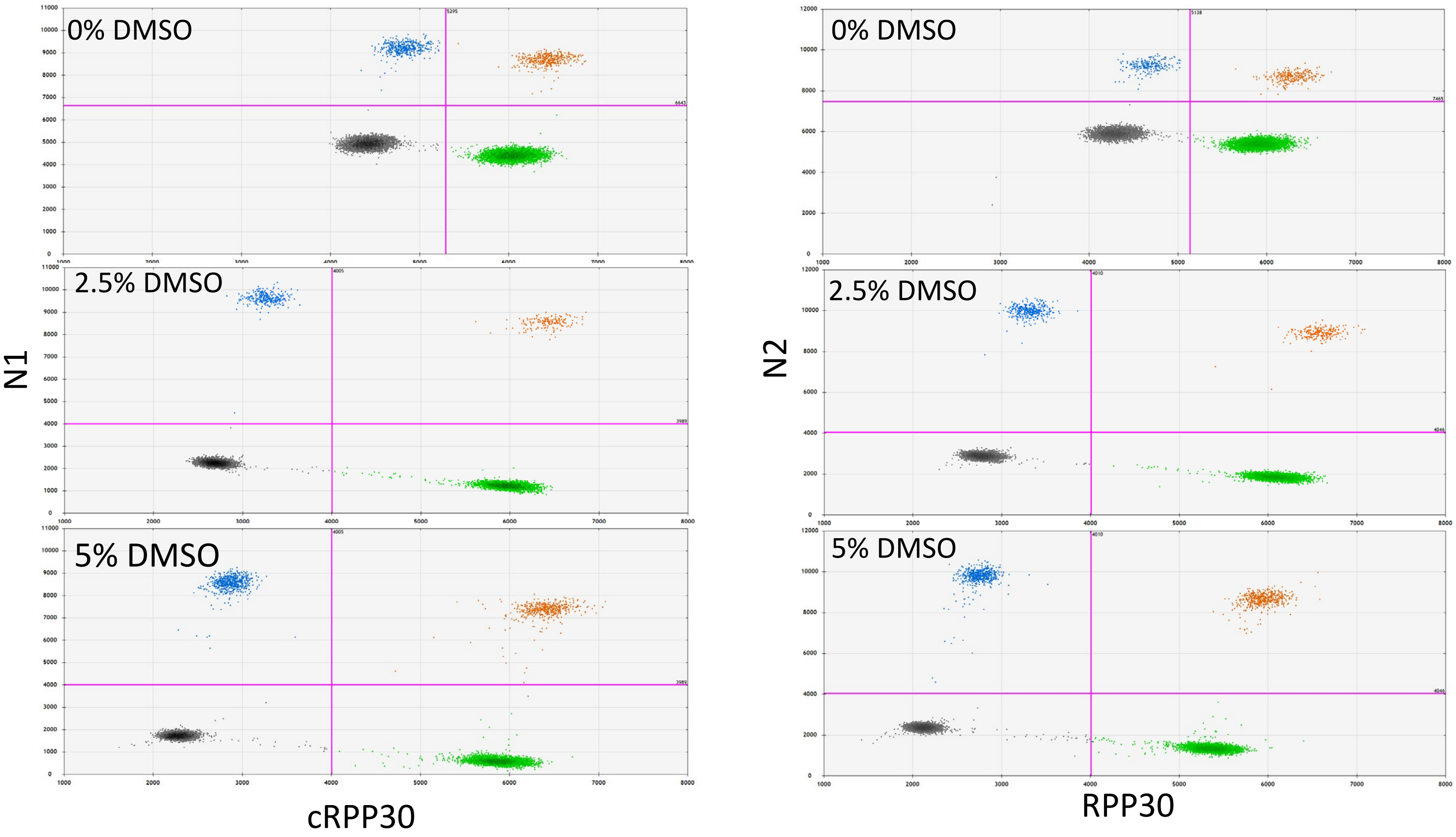

Figure S1. DMSO decreases negative droplet intensity in RT-ddPCR assays, related to Figures 1, 2, 4 & 5. The RT-ddPCR reactions containing 0%, 2.5%, or 5% DMSO were performed for a Chelex-lowTE sample prepared with the ATCC SARS-CoV-2 virions and 293FT cells using N1 and cRPP30 (left panel) or N2 and RPP30 (right panel). The grey clusters represent negative droplets; blue and green clusters represent Fam and Hex positive droplets, respectively; and orange clusters represent double positive droplets.

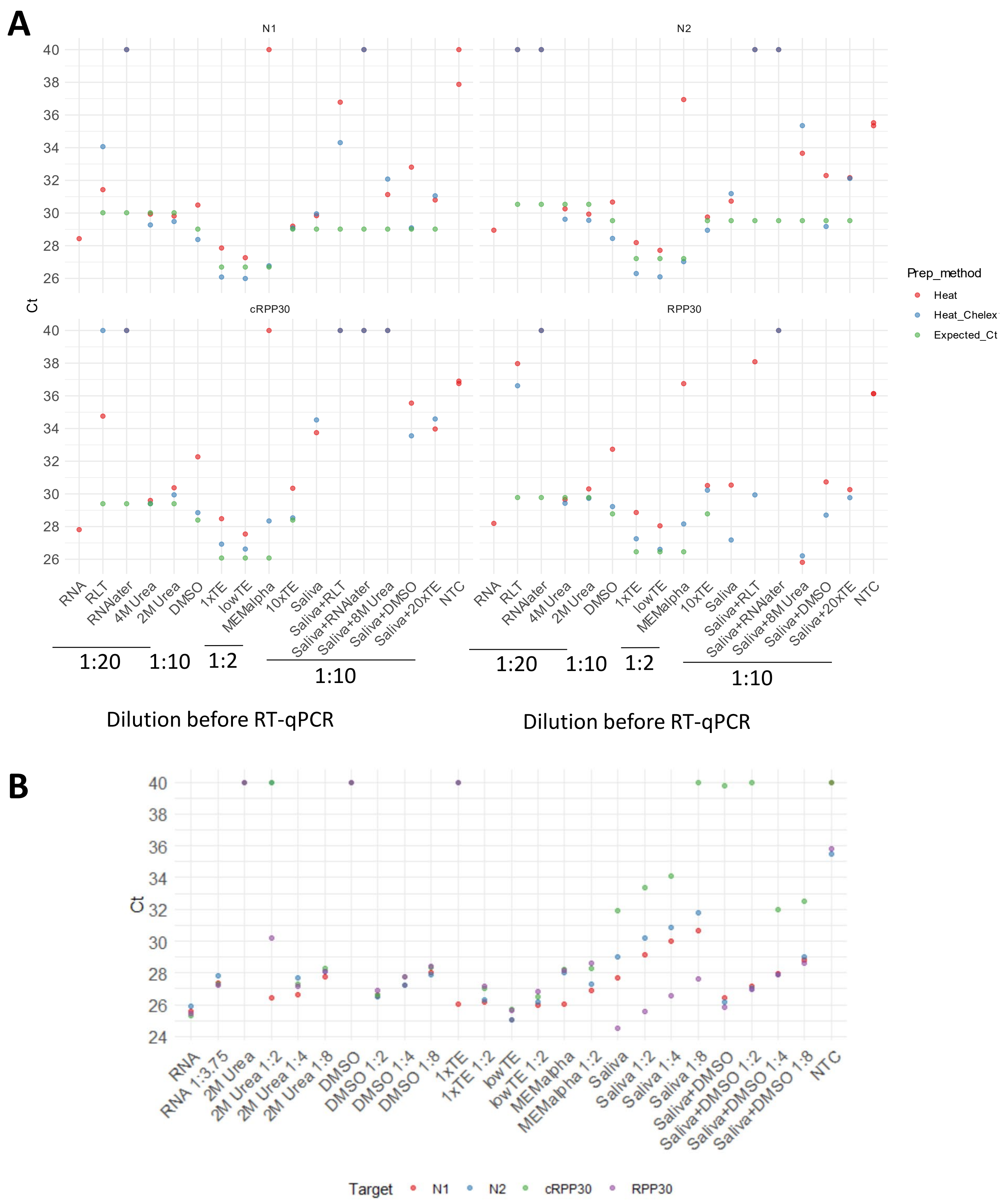

Figure S2. SARS-CoV-2 prepared in different buffers used for RT-qPCR without RNA-extraction, related to Figure 2. **(A)** Samples were diluted in H2O as indicated at the bottom. Expected_Ct refers to Ct calculated based on Ct from extracted RNA normalized with added virion numbers after dilution using the ΔCt method. **(B)** Buffer compatibility in RT-qPCR. Sample RNA, not heated and 5 μl of which contained materials extracted from 6,250 virions. Other samples were heated in the presence of Chelex, of which undiluted samples also contained 6,250 virions per 5 μl. Samples were diluted in H2O. Samples with undetermined Ct values were plotted as Ct 40. The NEB Luna RT-qPCR kit and NEB-Luna-Program I was used. NTC, no-template control.

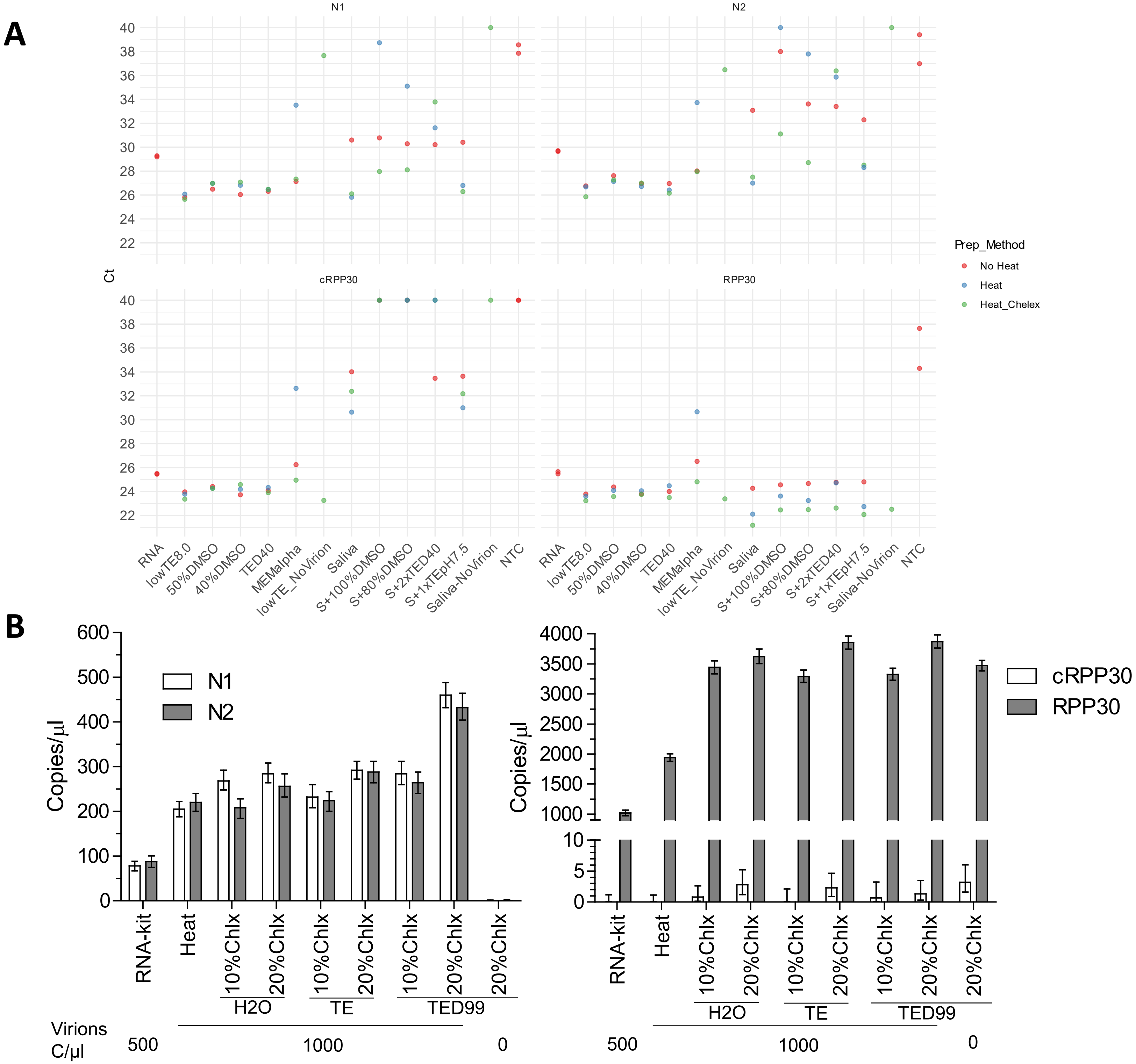

Figure S3. Tris EDTA and DMSO containing buffers, related to Figures 3, 4 & 5. **(A)** RT-qPCR of samples with heat-inactivated ATCC SARS-CoV-2 virions. 5 µl of samples were used for one reaction in RT-qPCR except that samples in MEM α were diluted 1:1 with H2O. "S+" refers to "Saliva + ". Samples with undetermined Ct values were plotted as Ct 40. The NEB Luna RT-qPCR kit and NEB-Luna-Program I was used. **(B)** RT-ddPCR of saliva samples with heat-inactivated ATCC SARS-CoV-2 virions. The Chelex was prepared in H2O, lowTE or TED99 (lowTE with 99% DMSO). RNA-kit refers to RNA extracted with the RNeasy Protect Saliva Mini Kit. NTC, no-template control.

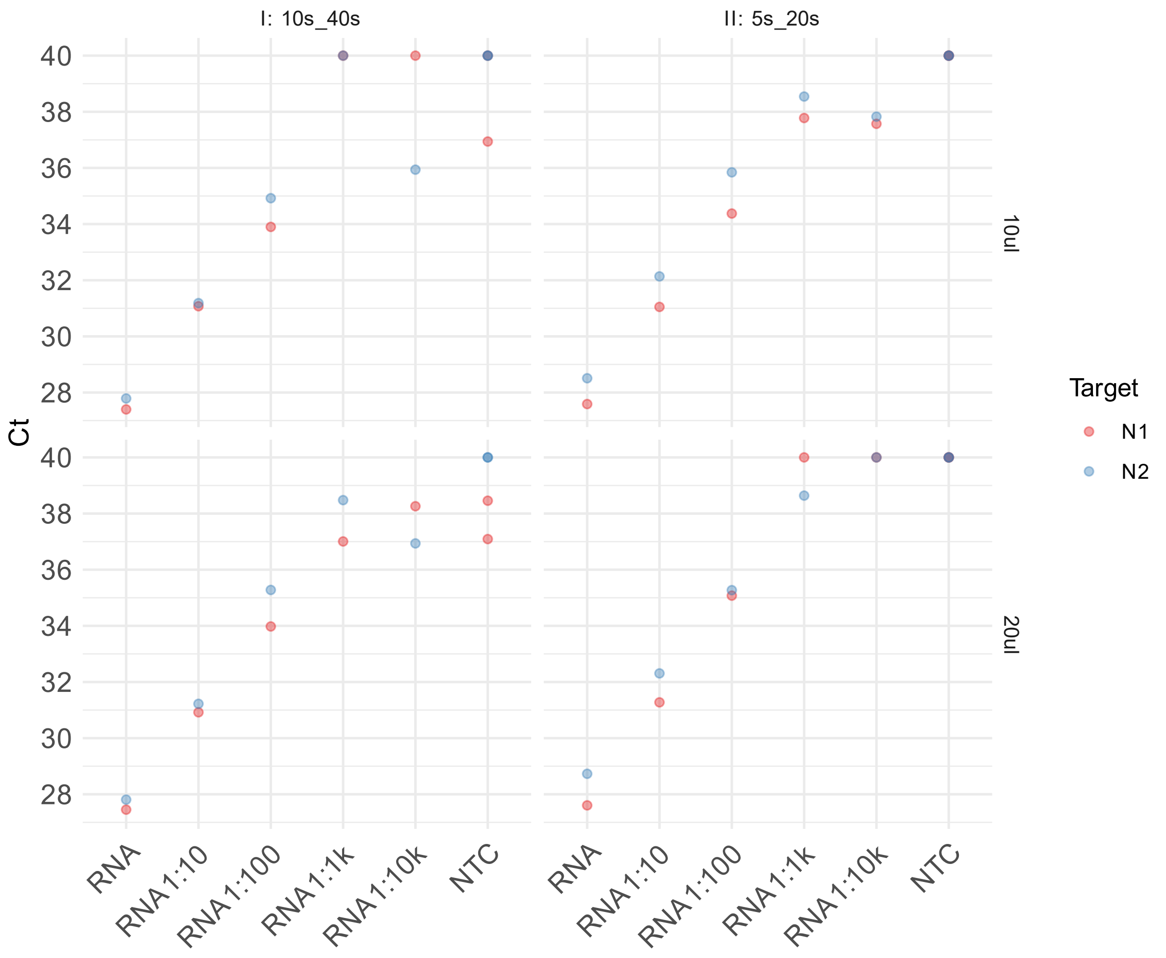

Figure S4. Optimization of the NEB Luna RT-qPCR assay, related to Figures 2 & 3. Extracted RNA samples were serial diluted and assayed either using 2.5 µl sample in a 10 µl reaction volume or 5 µl in a 20 µl reaction, and using a longer PCR protocol (**I**: 10 seconds of denature and 40 seconds of annealing/extension) or a shorter PCR protocol (**II**: 5 seconds of denature and 20 seconds of annealing/extension). NTC, no-template control.

Table S1. Oligonucleotides, related to STAR methods.

Target	Oligo name	Oligo sequence
2019-nCoV_N1	2019nCoV_N1F	GACCCCAAAATCAGCGAAAT
	2019nCoV_N1R	TCTGGTTACTGCCAGTTGAATCTG
	2019nCoV_N1Fam	ACCCCGCATTACGTTTGGTGGACC
2019-nCoV_N2	2019nCoV_N2F	TTACAAACATTGGCCGCAAA
	2019nCoV_N2R	GCGCGACATTCCGAAGAA
	2019nCoV_N2Fam	ACAATTTGCCCCCAGCGCTTCAG
RPP30 (aka RP)	RPP30F	AGATTTGGACCTGCGAGCG
	RPP30R	GAGCGGCTGTCTCCACAAGT
	RPP30Hex	TTCTGACCTGAAGGCTCTGCGCG
cRPP30	RPP30F	See above
	RPP30cR	GCAACAACTGAATAGCCAagGT
	RPP30Hex	See above
chr5UC	chr5UC-F	ATTTATGACCAGCCACAGCC
	chr5UC-R	CCATCAGGGACTTGGTTTCA
	chr5UC-Hex	CAACTCCAGCAGCTGCACACCGC
